# Supplementary material for: Predation and fragmentation portrayed in the statistical structure of prey time series
Source: BMC Ecol. 2009 May 6;9:10. doi: 10.1186/1472-6785-9-10 (PMC2689204; doi:10.1186/1472-6785-9-10)
Supplement: Additional file 2 — Voles and related classes ODDox Documentation. ODDox documentation of the agent-based model (ALMaSS) applied by Hendrichsen et al. The documentation is started by activating main.html. [file 1472-6785-9-10-S2.zip › Vole_ODDox/_vole_population_manager_8cpp.html]

ALMaSS ODDox: VolePopulationManager.cpp File Reference

- Main Page
- Related Pages
- Classes
- Files

# VolePopulationManager.cpp File Reference

---

## Detailed Description

**VolePopulationManager.cpp This file contains the source for the vole population manager class**   

by Chris J. Topping   
Version of 28th Jan 2001   
All rights reserved.   
  
With additions as noted in:   
April 2006   
November 2007   
Doxygen formatted comments in May 2008   

`#include "GeneticMaterial.h"`  
`#include <vector>`  
`#include "ls.h"`  
`#include "PopulationManager.h"`  
`#include "Vole_all.h"`  
`#include "Predators.h"`  
`#include "VolePopulationManager.h"`  
`#include "MovementMap.h"`  

|  |  |
| --- | --- |
|  |  |
| Defines | |
| #define | \_\_CIPEGRIDSIZE   500 |
| #define | \_\_SpecificPesticideEffectsVinclozolinLike\_\_ |
| #define | \_CRT\_SECURE\_NO\_DEPRECATE |
| Functions | |
| static CfgInt | cfg\_f1sterilitychance ("VOLE\_FONESTERILITYCHANCE", CFG\_CUSTOM, 0) |
| static CfgInt | cfg\_geneticproductfertilityeffect ("VOLE\_GENETICPRODUCTFERTILITYEFFECT", CFG\_CUSTOM, 50) |
| static CfgInt | cfg\_geneticsterilitychance ("VOLE\_GENETICSTERILITYCHANCE", CFG\_CUSTOM, 0) |
| static CfgInt | cfg\_GrassStartGrowth ("VOLE\_GRASSSTARTGROWTH", CFG\_CUSTOM, 4) |
| static CfgFloat | cfg\_MoveToLessFavourable ("VOLEMOVETOLESSFAVOURALBLE", CFG\_CUSTOM, 0.001) |
| static CfgInt | cfg\_vole\_starting\_number ("VOLE\_START\_NO", CFG\_CUSTOM, 2000) |
| Variables | |
| CfgBool | cfg\_CIPEGridOutput\_used |
| CfgFloat | cfg\_extradispmort |
| CfgInt | cfg\_pm\_eventfrequency |
| CfgInt | cfg\_pm\_eventsize |
| CfgBool | cfg\_ReallyBigOutput\_used |
| CfgBool | cfg\_ReallyBigOutputMonthly\_used |
| CfgBool | cfg\_RipleysOutput\_used |
| double | g\_extradispmort |
| double | MoveToLessFavourable |
| const float | ReproTable\_base [2][12] |

---

## Define Documentation

#define \_\_CIPEGRIDSIZE   500

Size of spatial grid output grids

Referenced by Vole\_Population\_Manager::TheCIPEGridOutputProbe().

#define \_\_SpecificPesticideEffectsVinclozolinLike\_\_

defined for work with Vinclozolin-like pesticides

#define \_CRT\_SECURE\_NO\_DEPRECATE

---

## Function Documentation

|  |  |  |  |  |
| --- | --- | --- | --- | --- |
| static CfgInt cfg\_f1sterilitychance | ( | "VOLE\_FONESTERILITYCHANCE" | , |  |
|  |  | CFG\_CUSTOM | , |  |
|  |  | 0 |  |  |
|  | ) |  |  | `[static]` |

Toxicological variable for specific pesticide effects

Referenced by Vole\_Population\_Manager::Init().

|  |  |  |  |  |
| --- | --- | --- | --- | --- |
| static CfgInt cfg\_geneticproductfertilityeffect | ( | "VOLE\_GENETICPRODUCTFERTILITYEFFECT" | , |  |
|  |  | CFG\_CUSTOM | , |  |
|  |  | 50 |  |  |
|  | ) |  |  | `[static]` |

Toxicological variable for specific pesticide effects

Referenced by Vole\_Population\_Manager::Init().

|  |  |  |  |  |
| --- | --- | --- | --- | --- |
| static CfgInt cfg\_geneticsterilitychance | ( | "VOLE\_GENETICSTERILITYCHANCE" | , |  |
|  |  | CFG\_CUSTOM | , |  |
|  |  | 0 |  |  |
|  | ) |  |  | `[static]` |

Toxicological variable for specific pesticide effects

Referenced by Vole\_Population\_Manager::Init().

|  |  |  |  |  |
| --- | --- | --- | --- | --- |
| static CfgInt cfg\_GrassStartGrowth | ( | "VOLE\_GRASSSTARTGROWTH" | , |  |
|  |  | CFG\_CUSTOM | , |  |
|  |  | 4 |  |  |
|  | ) |  |  | `[static]` |

The mean temperature over 14 days at which the grass is assummed to start to grow

Referenced by Vole\_Population\_Manager::DoFirst().

|  |  |  |  |  |
| --- | --- | --- | --- | --- |
| static CfgFloat cfg\_MoveToLessFavourable | ( | "VOLEMOVETOLESSFAVOURALBLE" | , |  |
|  |  | CFG\_CUSTOM | , |  |
|  |  | 0. | *001* |  |
|  | ) |  |  | `[static]` |

The probability of moving to less favourable habitats when walking

Referenced by Vole\_Population\_Manager::Init().

|  |  |  |  |  |
| --- | --- | --- | --- | --- |
| static CfgInt cfg\_vole\_starting\_number | ( | "VOLE\_START\_NO" | , |  |
|  |  | CFG\_CUSTOM | , |  |
|  |  | 2000 |  |  |
|  | ) |  |  | `[static]` |

How many voles to start with

Referenced by Vole\_Population\_Manager::Init().

---

## Variable Documentation

CfgBool cfg\_CIPEGridOutput\_used

Special spatial grid output used or not

Referenced by Vole\_Population\_Manager::Init(), and Population\_Manager::Run().

CfgFloat cfg\_extradispmort

Referenced by Vole\_Population\_Manager::Init().

CfgInt cfg\_pm\_eventfrequency

The frequency of catastrophes

Referenced by Vole\_Population\_Manager::Catastrophe().

CfgInt cfg\_pm\_eventsize

The size of catastrophes

Referenced by Vole\_Population\_Manager::Catastrophe().

CfgBool cfg\_ReallyBigOutput\_used

Raw data for spatial output for calculating Ripley's spatial statistics with additional information about habitat and physiology

Referenced by Vole\_Population\_Manager::Init(), Population\_Manager::Run(), and Population\_Manager::~Population\_Manager().

CfgBool cfg\_ReallyBigOutputMonthly\_used

Raw data for spatial output for calculating Ripley's spatial statistics

CfgBool cfg\_RipleysOutput\_used

Referenced by Vole\_Population\_Manager::Init(), Population\_Manager::Run(), and Population\_Manager::~Population\_Manager().

double g\_extradispmort

Referenced by Vole\_Population\_Manager::Init().

double MoveToLessFavourable

This is specified as a global because it avoids costing time on look-ups.   
This is needed because of the extreme CPU intensive movement functions

Referenced by Vole\_Base::DoWalking(), Vole\_Base::DoWalkingCorrect(), and Vole\_Population\_Manager::Init().

const float ReproTable\_base[2][12]

**Initial value:**

```
 { {0,0,0,0,4,4,4,4,3,2,3,0},
                                 {0,0,4,4,5,5,5,5,4,4,0,0} 
                               }
```

Litter sizes dependent upon month and year

Referenced by Vole\_Population\_Manager::Init().

---

Generated on Thu Jan 22 14:13:45 2009 for ALMaSS ODDox by 
 1.5.6
